# Dental Educational Methods

**Published:** 1900-02

**Authors:** 


### Editorial.

### DENTAL EDUCATIONAL METHODS.

THE recent meeting in Philadelphia of the National School of Dental Technics naturally has led educators in dentistry to consider the grave problems that surround dental training at the present time and the prospect for the future.

The meeting, which was largely attended from all parts of the country, gave evidence, not only through the exhibits, but from the papers read and discussions thereon, that there was a healthy determination to seek out the best methods for dental education, and to spare neither time nor money in their accomplishment. There is no need for discouragement in college work, when men are willing to cross the continent to spend three days with their fellow-workers over the problems of the curriculum, and how best to overcome the difficulties environing this work.

The dental colleges have been exposed to a continued storm of criticism from those not conversant with the work, and it would seem that these detractors might receive, in this unselfish devotion, an object lesson that ought to cause them to retire to everlasting silence. That this will be the result is scarcely to be hoped for; certainly not until the old generation, who regard the laboratory as the sum total of dental educational excellence, has passed away.

The National School of Dental Technics, like many other organizations, began its life in somewhat crude conceptions of its work; in fact, its attitude towards dental education had the effect of driving many away from it, and this feeling with some still continues. Experience has changed this, and has led up to a modification of name to cover the broader conception of its duties and responsibilities. It is this departure from the original narrow conceptions that ought to attract the co-operation of all dental educators. An association is needed in our profession where there can be a free interchange of thought as to the best methods of training under-graduates in our colleges. Dental educators naturally adopted methods that had been in use in medical teaching for many centuries, and at the beginning these were satisfactory. The time came, however, when it was apparent that new methods must be adopted. The curriculum expanded and became a burden, and its re-arrangement a necessity. This led to the organization of the National Association of Dental Faculties, with the hope that all difficulties would be met and conquered through interchange of views. Sixteen years have passed since that original meeting, and the work of that body stands out prominently as the most important factor in dental educational progress. It can be stated without fear of successful contradiction that the results of its efforts have been of more value than all others in elevating the standard, unifying the work, and establishing dental education upon a solid basis for the future. Its work has been, however, mainly of a legislative character, and has not advanced materially the internal educational forces in the several schools under its jurisdiction. This has not been in accord with the original conception of its founders. It was supposed that three or four years would accomplish, practically, all that might be desirable in this direction, and that the weightier problems of student-training would receive due consideration. Up to this period this has not been done, and the "Faculties" is still yearly struggling with questions that have largely ceased to have general interest. The fact that it seemed impossible to secure proper consideration of questions in dental pedagogy led to the formation of the School of Dental Technics.

The question that now interests the writer, and, perhaps, many others, is, why there should be two distinct associations, avowedly with the same object and composed of the same men? To call two associations together, thus composed, at a large expenditure of time, money, and vitality, seems to be a waste of energy. If this were confined to a small territorial area there might be a value in separating the work, but when a continent, embracing thousands of miles is considered, it does not seem to be a wise method of procedure.

If the School of Technics is to be continued as a separate organization, it would seem that the best course to adopt would be a meeting once in three years. The reason for this proposed change must be apparent. One year is too short a period to effect any marked results in methods of work. The members come together and listen to papers and discussions that, in the main, reiterate the ideas of the previous years. In time this grows monotonous, and interest flags and the organization eventually dies.

The better plan would be to amalgamate the two organizations, either forcing the Association of Faculties into the School of Technics, or the latter into the former, thus combining the work of both and materially strengthening both associations, and that without sacrificing the work of either.

It must be evident to those interested that the work of the Association of Faculties has not been entirely satisfactory of recent years. The time of this body has been largely occupied in considering matters that should have been disposed of in other ways, and have thus allowed the members time to consider educational problems.

It is very clear that if the Association of Faculties does not quickly move to a broader comprehension of its legitimate work, the time cannot be far distant when those schools that have little to gain by continuing in membership will lose all interest. The higher schools cannot affiliate with those who continue to adopt the political methods so openly made use of at Niagara and the meeting the preceding year. Their interest lies in maintaining a standard that will give a higher scholarship and, through this, an added dignity to dental character.

The next session of this body must certainly meet the demand for more time. It is either four years, or a reduction in the demands of the curriculum. It must be evident to all experienced teachers that we are graduating men of partial culture in many things and experts in nothing. The practical branches are suffering as never before, and this in spite of the time devoted to technical work. The Association of Faculties raised the entrance requirements to the second year of a high school, and some of the schools require a diploma from such a school. It is too early to express an opinion as to the value of this higher entrance standard from the practical side, but from present indications dentistry will have in the future a body of men of broader intelligence, but inferior in those qualities that make the practical dentist.

The time of the meeting at Old Point Comfort in June next should not be taken up in considering unimportant matters, but should be mainly devoted to the higher questions that this article has endeavored briefly to point out. It is for the men connected with the two educational organizations to calmly consider the difficulties that lie in the path of the earnest college educator, and this can better be accomplished by a united effort and a reorganization upon broader lines than has heretofore been regarded as essential.

